# Analyse ergonomique semi-quantitative des contraintes biomécaniques du rachis cervical parmi les opérateurs sur écran dans les établissements universitaires tunisiens

**DOI:** 10.11604/pamj.2018.31.189.12474

**Published:** 2018-11-19

**Authors:** Amira Omrane, Olfa Jlassi, Salma Kammoun, Ines Tka, Awatef Kraiem, Mohamed Adnène Henchi, Taoufik Khalfallah, Lamia Bouzgarrou

**Affiliations:** 1 Département de Médecine de Travail et d’Ergonomie, Faculté de Médecine de Monastir, Tunisie

**Keywords:** Cervicalgies, travail sur écran, contraintes biomécaniques, ergonomie, Neck, on-screen work, biomechanical constraints, postural and gestural, ergonomics

## Abstract

**Introduction:**

Evaluer les contraintes biomécaniques associées aux cervicalgies parmi les opérateurs sur écran.

**Méthodes:**

Etude ergonomique réalisée au prés de 325 opérateurs sur écran dans 25 établissements de l’université du centre tunisien, basée sur une étude anthropométrique du poste du travail sur écran et une analyse semi-quantitative sur des enregistrements vidéo des activités des travailleurs conduits sur une période représentative de 30 minutes.

**Résultats:**

La prévalence des cervicalgies évaluée à 72,3%, a concerné une population de travailleurs qui exerçait avec un siège et un plan de travail inadaptés (84,6%) et un écran bas situé par rapport au regard horizontal (81,2%). L’analyse semi-quantitative par enregistrement vidéo du poste de travail a conclu que la nuque était maintenue en flexion franche de plus de 40 degrés sur 69% du temps global du travail. La flexion latérale a été visible sur 50,3% du temps de travail et la rotation droite ou gauche sur 57,4% du temps de travail. Ainsi, Les opérations de prise d’information de l’écran, de regard du clavier et de consultation des documents se sont dégagées comme les plus de contraignantes pour la nuque sur le plan gestuel et postural.

**Conclusion:**

Nos résultats mettent en évidence l’importance des contraintes biomécaniques associées à la conception du poste de travail sur écran non conforme aux dimensions anthropométriques ergonomiquement recommandées. Ces contraintes sont associées à une prévalence élevée des cervicalgies traduisant la nécessité d’aménagement ergonomique de ces postes de travail pour prévenir ce fléau.

## Abstract (en)

**Introduction:**

This study aims to evaluate the biomechanical constraints associated with neck pain among screen-operators.

**Methods:**

We conducted an ergonomic study of 325 screen-operators in 25 Tunisian universities based on an anthropometric evaluation of on-screen work and on a semi-quantitative analysis of video recordings of the activities of employees over a representative period of 30 minutes.

**Results:**

The prevalence of neck pain was 72.3% in a working population who had to seat in an unsuitable chair and to use an unsuitable worktop (84.6%) and a screen too low with regard to horizontal gaze (81.2%). A semi-quantitative analysis, using video recording of the workstation, concluded that the nape flexed more than 40 degrees for 69% of overall working time. Lateral flexion was observed for 50.3% of working time and right or left rotation for 57.4% of working time. Thus, the most limiting head posture on a postural and gestural level was observed when screen-operators read on a computer screen, had an incorrect positioning of the keyboard and consulted documents.

**Conclusion:**

Our results highlight the importance of biomechanical constraints associated with a design of on-screen work which is not in accordance with the ergonomically recommended anthropometric dimensions. These constraints are associated with a high prevalence of neck pain, reflecting the need to ensure an ergonomic design of workstations in order to prevent this problems.

## Introduction

Les cervicalgies sont un problème fréquent en milieu de travail. En effet, près des deux tiers des travailleurs endurent ces algies à un moment de leurs vies [1]. De plus, les cervicalgies génèrent un coût élevé pour l'individu et pour la société [1-3]. Comparés aux autres professions, les travailleurs sur écran occupent la prévalence annuelle la plus élevée de ces troubles (variant entre 17,7 et 63%) [1, 4, 5]. Il est actuellement admis que les cervicalgies sont des pathologies multifactorielles [1]. Reste que selon les données de la littérature, contraintes biomécaniques associées au travail sur écran se sont des forts prédicateurs du développement de ces plaintes [1, 6, 7]. L'actuel travail constitue une deuxième étape d'une étude initiée à la demande du service de médecine de travail des établissements universitaires au centre tunisien. De ce fait, la première partie cette étude a consisté en une enquête transversale conduite sur trois mois auprès des salariés des 25 institutions des divers pôles universitaires du centre tunisien et a permis d'estimer la prévalence des cervicalgies chez ces opérateurs et d'identifier leurs facteurs de risque sociodémographiques et professionnels [8]. En complément, la deuxième partie de l’étude- présentée dans cet article, est basée sur une évaluation ergonomique du poste du travail sur écran, suivie d’une analyse semi-quantitative des activités des travailleurs réalisées sur des enregistrements vidéo conduits sur une période représentative de l'activité. L’objectif de cette partie du travail a été d'évaluer les contraintes biomécaniques associées à la genèse des cervicalgies.

## Méthodes

### Population d’étude

La population d’étude a inclus l’ensemble des opérateurs manipulant l’écran de visualisation depuis au moins un an, identifiés sur la base des listes nominatives fournies par les services de gestions de ressources humaines et précisant les dates et postes d’affectation. Au total, 325 opérateurs sur écran, majoritairement masculin (sexe ratio: 1.3) et d'un âge moyen de 39,93 ± 7,34 ans (extrêmes égales à 21 et 60 ans) ont été inclus. L’ancienneté moyenne du travail sur écran a été de 8,3 ± 2,9 ans. Les postes les plus occupés ont été les postes de secrétaire (42,7%), suivi par celui de technicien supérieur et d’administrateur. Parmi ces opérateurs, 72,3% des sujets, répondaient à la définition de cervicalgiques qui renvoi à « tout sujet ayant présenté, au cours des 12 derniers mois précédent l’enquête, une douleur à point de départ cervical et de durée minimale de 12 heures » [8].

La démarche adoptée dans cette étude ergonomique a consisté en bref à: identifier, sur la base des données de l'observation directe, une période de travail représentative; décomposer les tâches du travail en opération élémentaires; réaliser des enregistrements vidéo en temps réel durant la période représentative; évaluer pour chacune des opérations élémentaires le niveau de force; réaliser des observations instantanées, par arrêt sur image sur ces enregistrements, en encadrant l’opération effectuée ainsi que les catégories de posture de la zone corporelle concernée.

Ainsi, nous avons procédé, dans un premier temps, à des observations directes pour mieux comprendre l’activité des opérateurs, les conditions d’exposition, détailler les principales catégories de tâches effectuées et identifier les opérations élémentaires composant chacune de ces tâches. Ces observations directes conduites sur une période minimales de 2 heures à chaque poste, ont concerné les 325 opérateurs des 25 institutions universitaires inclus dans l'étude. Elles ont permis d'évaluer les caractéristiques de l’interface homme-machine, notamment les contraintes angulaires, ainsi que l’ambiance lumineuse au poste par un luxmètre équipé d’un détecteur photodiode. En se basant sur les données de cette première phase d’observation directe, un échantillon représentatif par groupes d’expositions homogènes(GEH) a été construit de façon aléatoire [9]. Un GEH, est défini comme un ensemble de travailleurs partageant le même profil d’exposition vu la similarité des déterminants en jeu (exp. l’environnement, l’emploi, les tâches exécutées…) [10]. En complément, des enregistrements vidéo ont été réalisés sur une durée représentative de l'ensemble de l’activité du travail auprès les opérateurs de l‘échantillon. Ces enregistrements ont ciblé la zone cervicale identifiée comme étant la plus à risque et ont permis de caractériser l’exposition moyenne des opérateurs susceptible d’entraîner une pathologie à court ou à moyen terme. L’analyse semi-quantitative des exigences biomécaniques de ces postes s'est basée sur le logiciel ERGOROM permettant notamment un découpage des séquences vidéo à des intervalles de temps réguliers, en 100 arrêts sur image pour assurer le codage des positions articulaires (de la nuque dans notre cas) selon les trois axes corporels et leur analyse selon la technique décrite par Indesteege et Malchaire [11].

Deux indices pu être calculés: un indice de variabilité gestuelle allant de 0(aucune variabilité gestuelle) à 1 (variabilité gestuelle importante), calculé en fonction de nombre du changement de position par unité de temps; un indice de niveau de force déployée au cours des opérations élémentaires calculé selon l’échelle de BORG [12].

## Résultats

### Données de la phase d’observation

Cette phase a permis de dégager le travail sur écran comme étant la composante principale de l’activité de 81,5% des opérateurs. Parmi les sujets observés, 92,5% travaillaient trois heures ou plus devant l’écran. Concernant le niveau de l'éclairage, il était insuffisant (< 300Lux) dans 71,1% des cas. De plus, l’étude des caractéristiques anthropométriques de l’espace du travail et des interfaces homme-machine a objectivé une non-conformité aux normes ergonomiques dans la majorité des cas (Tableau 1). Les cervicalgies étaient significativement plus fréquentes quand la hauteur du dossier de siège était inférieure à 50cm (p = 0.004), la hauteur assise–sol entre 42 et 55cm (P = 0.007) et l’hauteur du plan de travail dépassait 75cm (P < 0.001). Par ailleurs, une augmentation statistiquement significative des cervicalgies était notée en cas de distance œil - écran inférieure à 50 cm (p < 10^-3^), de distance œil - document en dehors de la zone 50 à 70cm (p = 0.01), d'angle de regard à l'écran inférieur à 10° (p < 10^-3^), d'angle de posture des membres inférieurs inadéquat avec profondeur pour les pieds insuffisante ( p < 10^-3^) et aussi en cas d'un local insuffisamment éclairé (p < 10^-3^). L’observation fine de l’activité sur écran proprement dite des opérateurs a permis de mettre en évidence cinq tâches principales: saisie des données (T1): la transmission de données généralement issues de documents imprimés ou manuscrites à l’ordinateur par l’entremise du clavier; acquisition des données(T2): la demande d’information d’une banque de données pour les utiliser dans le cadre d’un dossier ou pour fournir des renseignements; communication interactive (T3): c’est un dialogue avec l’ordinateur de type question réponse; traitement du texte (T4): cette tâche consiste à introduire, structurer la présentation et corriger les textes initialement imprimés ou manuscrits; tâches spécifiques (T5): regroupant un ensemble d’actions réalisées tel que la programmation, illustration par graphisme, manipulation de logiciels spécialisés.

**Tableau 1:** Données de l'étude anthropométrique du poste de travail et de l'interface homme-ordinateur

Caractéristiques du poste de travail sur écran (Norme)		Effectif	%
Caractéristiques du poste de travail sur écran	***Siège:***		
	Hauteur assise-sol (42-55)	265	81,5
	Hauteur du dossier (=50)	10	3,1
	***Plan de travail:***		
	Hauteur touche proximale-sol (65-75)	69	21,2
Caractéristiques de l'Interface Homme-Ordinateur	Distance œil – écran (50 - 70 cm)	120	36,9
	Distance œil – document (50 - 70 cm)	270	83,1
	Position écran / Regard horizontal (10-20°sous horizon)	42	12,9
	Angle bras-avant-bras 90°	92	28,3
	Angle cuisse /Jambe (90°)	92	28,3

Lors de l’exécution de l’ensemble de ces tâches, quatre opérations élémentaires centrales ont été observées (Op1-4), avec deux sous-tâches intermittentes interférant avec le travail sur écran (Op 5-6). Ces opérations élémentaires sont : la prise d’information sur écran (Op1), le regard du clavier (Op2), la lecture ou la consultation des documents (Op3), la manipulation de l’imprimante (Op4), la communication téléphonique (Op5), la communication avec une tierce personne nb (Op6).

### Les données de l’analyse semi-quantitative

Conformément aux données de la phase d’observation et celle des études de poste, les conditions d’exposition au travail sur écran (environnement et ambiances physiques, conception des espaces..) étaient globalement comparables dans les différents institutions, pour l’ensemble des opérateurs et ce, quel que soit le poste occupé ou la fonction assurée au sein des équipes respectifs. Ainsi, cette population d’étude peut être considérée comme un GEH unique. Conformément au tableau d’échantillonnage des GHE, un tirage aléatoire de 15 opérateurs a été pratiqué pour identifier les sujets à enregistrer [9]. Pour chacun de ces sujets de l’échantillon, un enregistrement vidéo, focalisé sur la nuque, a été réalisé sur 30 minutes: durée identifiée lors de la phase d’observation comme un intervalle de stationnarité, suffisamment long pour être représentatif de l’ensemble de l’activité des opérateurs. A l’aide du logiciel ERGOROM, ces enregistrements ont fait l’objet d’une analyse semi- quantitative avec des arrêts réguliers sur image espacés de 18 secondes. Au total, sur chaque enregistrement, cent images ont été codées, selon la position du cou dans les plans transversal et sagittal (Tableau 2). L'analyse a notamment conclu que la nuque est maintenue en flexion franche de plus de 40 degrés sur 69% du temps global du travail, avec une variabilité modérée du mouvement (indice de variabilité = 0.4%). De plus, la flexion latérale a été visible sur 50,3% du temps de travail et la rotation droite ou gauche sur 57,4% de ce temps. Les opérations de prise d’information de l’écran, de regard du clavier et de consultation des documents se sont dégagées comme les plus de contraignantes pour la nuque sur le plan gestuel et postural. En effet, l’opération de prise d’information (Op1), qui a occupé 43,8% du temps global du travail, s’est exercée durant 60,3% de son temps en flexion franche de la nuque, alors que l’opération du regard du clavier (Op2) qui n’occupait que 16,4% du temps global, s’est exercé dans 96,7% de son temps en flexion franche. L’opération de consultation d’un document (Op3) qui a occupé 19,9% du temps de travail a été effectuée respectivement en flexion franche de la nuque, en rotation latérale et en flexion cervicale latérale dans 96,3%; 74,2% et 59,5% de son temps Par ailleurs la force moyenne déployée a été estimée à 0,5 correspondant à un niveau « d’effort faible » selon l’échelle de Borg. Les indices de variabilité gestuelle ont été qualifiés de faible pour les mouvements de rotation (indice = 0,37) et de modéré dans les mouvements de flexion/ extension et de flexion latérale (indices = 0,45).

**Tableau 2:** La répartition des opérations en fonction du temps global, la position de la nuque selon les trois axes spacieux et le niveau de force selon l’échelle de BORG

	Opérations						Total	Valeurs extrêmes (%)
	1	2	3	4	5	6		
Durée (% temps)	43,8	16,4	19,4	5,6	5,2	9,1	100
**Flexion/extension**								
Neutre 1	17,4	0,5	0,7	3,7	3	5,7	31	15 - 43
Flexion franche	26,4	15,9	19,2	1,9	2 ,2	3,4	69	57 - 85
**Flexion latérale**								
Neutre	26,9	7,6	8,1	3,5	0,8	2,8	49,7	30 - 71
Flexion latérale:								
visible (à droite 1								
ou à gauche) i	16,9	8,8	11,8	2,1	4,4	6,3	50,3	29 - 70
**Rotation**								
Neutre	28,3	8,2	5,1	0	0,7	0,3	42,6	9 - 58
Rotation visible (à droite ou àgauche)	15,5	8,2	14,8	5,6	4,5	8,8	57,4	42 - 81
**Force**	**Score de BORG**						Moy	
	0,5	0,5	0,5	0,5	0,5	0,5	0.5	
**Répétitivité**							Indice	
Flexion / Extension							0,40	0.16 - 0.56
Flexion latérale							0,45	0.31 - 0.57
Rotation							0,37	0.16 - 0.46

Opération 1: la prise d’information sur écran, Opération 2: le regard du clavier, Opération 3: la lecture ou la consultation des documents, Opération 4: la manipulation de l’imprimante, Opération 5: la communication téléphonique, Opération 6: la communication avec un visiteur.

## Discussion

L’actuelle étude ergonomique a fait suite à une enquête transversale conduite auprès des 325 opérateurs des institutions universitaires du centre tunisien manipulant l’écran de visualisation depuis au moins un an. Cette première partie, a objectivé, en se référant au questionnaire Nordique, une prévalence des cervicalgies sur les 12 derniers mois précédents l’enquête de 72,3% [8]. L’actuel travail a été conduit en se basant, dans un premier temps, sur l’observation directe de l’ensemble des opérateurs et l'évaluation anthropométrique des postes de travail et des caractéristiques de l’interface homme-machine. Cette première phase d’observation directe a permis d’identifier cinq différentes tâches avec globalement six opérations élémentaires: prise d’information sur écran, regard du clavier, lecture des documents, manipulation de l’imprimante, communication téléphonique et communication avec un visiteur. De plus, cette première phase a permis de conclure que les conditions de travail et les activités pratiquées par l’ensemble des opérateurs sont globalement similaires, définissant ainsi un groupe d’exposition homogène. Ainsi pour l’analyse semi-quantitative, les enregistrements vidéo ont été réalisés auprès d’un échantillon composé de 15 opérateurs, conformément au tableau d’échantillonnage des GHE [9]. Chaque enregistrement a été conduit sur 30 minutes de travail effectif, durée jugée représentative de l'ensemble de l’activité du travail.

L’analyse ergonomique semi-quantitative par observation, inspirée de la méthode OWAS, a comme objectif de caractériser l’exposition moyenne des opérateurs et d'évaluer l’intensité des contraintes biomécaniques auxquelles est soumise la zone corporelle atteinte à savoir le rachis cervical. Reste que les enregistrements vidéo peuvent influencer les comportements des personnes observées, en induisant soit plus d’aléas gestuels, soit au contraire, être associé à un comportement plus normatif, avec moins de mouvements collatéraux, plus de temps de pauses… Par contre, les enregistrements vidéo proposent un avantage principal qui est le confort d’analyse avec possibilité d’arrêt sur image permettant à l’observateur de prendre son temps avant de décider le score à attribuer à la posture observée. Afin de réduire le biais observateur, les enregistrements ont été pratiqués par un ergonome expérimenté qui s’est positionné en retrait avec explication de l’intérêt de ces observations. L’étude anthropométrique des postes de travail a conclu au non respect des normes ergonomiques avec, notamment, 85% des sièges inadaptés. Le siège est une composante principale du poste de travail, non seulement pour le travail sur écran, mais aussi pour toute activité professionnelle exécutée totalement ou partiellement en position assise. De plus, le dossier de la chaise, constitue l’élément central d'un siège de travail puisqu'il est doté d'une fonction de soutien importante pour la région cervicale et les disques intervertébraux [13]. Plusieurs études se sont intéressées à l'étude biomécanique à la fois du siège et du poste de travail avec des résultats qui étaient parfois divergents [14-17]. En effet, Yu It *et al.* [18] avaient rapporté une association significative entre une hauteur inadaptée de la chaise et la prévalence des cervicalgies. Par ailleurs, Ignatius YT *et al.* [15] avaient établi une relation entre la survenue de cervicalgie et l'utilisation des chaises à hauteur non réglable (OR = 2,83). Toutefois, Cail F *et al.* [13] ont rapporté qu'un siège sans dossier ou avec un dossier incliné de vingt degrés était moins confortable qu'un siège classique. En effet, lorsque l'inclinaison du dossier vers l'arrière augmente, la pression sur les disques intervertébraux, ainsi que l'activité électrique des muscles des régions cervicales, thoraciques et lombaires, diminuent [13]. D'autre part, Visser B *et al.* [19] avaient conclu, que seuls les opérateurs utilisant des sièges avec accoudoirs, avaient une activité réduite des muscles de cou et des épaules, ce qui améliorait la tension des muscles cervicaux et diminuait les plaintes scapulo - cervicales. De plus, Marcus M *et al.* [20] et Bergqvist U *et al.* [14] avaient noté une association significative entre l'absence des accoudoirs au niveau des chaises et la présence des douleurs cervicales et ¬scapulaires. Dans notre étude, la conformité par rapport aux normes des angles bras-avant bras, n'était satisfaisante que pour 28,30% des opérateurs sur écran. De plus, une corrélation entre la cervicalgie et un angle bras avant - bras non conforme a été objectivée (p < 10^-3^). Contrairement à nos résultats, plusieurs études [18, 21] n’avaient pas noté de cette corrélation entre les cervicalgies et un angle bras avant - bras inadéquat. En effet, Waersted M *et al.* [21], lors d'une étude expérimentale, n’avaient pas trouvé de corrélation significative entre l'angle bras - avant bras et l'activité du muscle trapèze et des muscles du cou, ainsi avec la survenue de cervicalgies. De même, Yu lt *et al.* [18], n'avaient pas noté d'association significative entre la cervicalgie et la position des bras par rapport aux avant-bras, ainsi que la posture statique et les mouvements répétés des bras.

Dans notre étude nous avons retenu qu'une hauteur touche proximal-sol trop importante était un facteur de risque de cervicalgie (p < 10^-3^). De nombreuses études confirment nos résultats [14, 22-24]. En effet, un clavier situé à une hauteur conforme aux normes ergonomiques (entre 65 et 75cm), diminueraient de façon considérable et significative la charge des muscles du cou réduisant ainsi les cervicalgies [25]. Par ailleurs, dans la majorité des opérateurs dans notre série (81,2%), travaillaient avec un écran bas situé. Cette conception était un facteur prédictif des cervicalgies (p < 10^-3^). Ce résultat a été confirmé par les résultats de l'analyse semi ¬quantitative par enregistrement vidéo. En effet, la nuque a été maintenue en flexion franche plus des deux tiers du temps global du travail, avec une corrélation entre la prévalence des cervicalgies et la flexion du rachis cervical. Par conséquent, l'opération de prise de l'information de l'écran, qui s'effectuait dans 60% de son temps en flexion cervicale franche, s'est dégagée comme étant l'opération élémentaire majeure. Une multitude d'études relatives au travail sur écran avaient analysé le positionnement de l'écran [1, 26-34]. Ces études avaient conclu que le travail moyennant un écran bas situé avec nuque fléchie ou haut situé était à l'origine de cervicalgies [1, 31-34]. Ainsi Burgess - Limerik R *et al.* [35, 36], avaient montré que le travail sur un écran situé au même niveau du regard horizontal, améliorait l'inconfort musculaire. De plus, Ariëns *et al*. [37] ont retrouvé un excès de risque d’apparition des douleurs cervicales parmi les opérateurs travaillant avec le cou fléchi au minimum à 20° pendant plus de 70% du temps de travail .Dans une étude ultérieur, cette même équipe a prouvé qu’un travail avec un cou fléchi au minimum de 45 degrés durant plus de 25% du temps de travail est associé à un RR = 2.2 de développer des cervicalgies [38]. Toutefois, Aaras A *et al.* (1997) [25], Yu It *et al.* (1996) [18], n'avaient pas trouvé de différence dans le positionnement de l'écran par rapport au regard horizontal et la genèse des cervicalgies.

Dans notre étude une distance œil - document ou œil - écran insuffisante a constitué un facteur de risques des cervicalgies (p respectivement de 0,01 et < 0,0001). Jaschinski W *et al.* [39] avaient noté qu'une distance œil - écran très importante était responsable de TMS de la nuque et des épaules. De plus, l'opération de consultation d’un document (Op3) a occupé 19,9% du temps de travail et s'est effectuée respectivement en flexion franche de la nuque, en rotation latérale et en flexion cervicale latérale dans 96,3%; 74,2% et 59,5 % de son temps. En effet, la posture adoptée et les mouvements effectués lors de l’exercice de cette opération tel que la rotation latérale sont fortement dépendants de l’aménagement de l’espace du travail en particulier le positionnement du document source, avec ou sans port document. Alors que Ariëns *et al*. [37], avaient conclu qu’une rotation du rachis cervical avec un angle = 45° maintenu sur plus de 30% du temps de travail n'est pas associée à un excès de risque la douleur cervicale à l’analyse uni ou multi variée (RR brut de 0,86 et RR ajusté de 0,98). Bauer W *et al.* [40] avaient signalés que le porte document devrait être placé à côte de l'écran pour et sur le même niveau, afin de minimiser les mouvements de flexion latérale et d'inclinaison de la tête lors de la consultation simultanée d'écran et du document.

Dans notre étude, la tâche de saisie des données implique l’opération de prise d’information (Op1), l’opération du regard du clavier (Op2) et aussi l'opération de lecture ou la consultation des documents (Op3) couvrant ainsi plus de la moitié du temps global de travail. Les données de la littérature affirment que la tâche de saisie (plus de 30 heures par semaine) est plus pourvoyeuse de cervicalgies [41]. Dans notre étude, Les indices de variabilité gestuelle ont été qualifiés de faible pour les mouvements de rotation (indice = 0,37) et de modéré dans les mouvements de flexion/ extension et de flexion latérale (indices = 0,45). L'absence de variabilité des tâches lors du travail sur écran s'est révélé être un facteur de risque de cervicalgies (méta RR = 1,27) [1]. Enfin, dans notre étude, nous avons trouvé qu'un local insuffisamment éclairé constituait un facteur de risque des cervicalgies (p < 10^-3^). Ce résultat s'accordent avec les études de Aaras A *et al.* [42, 43], Korhomen T. [23] qu’un éclairage insuffisant constitue un facteur de risque de cervicalgies.

## Conclusion

Les cervicalgies constituent une plainte fréquente en milieu de travail, notamment parmi les opérateurs sur écran, avec un coût direct (le coût du traitement) et indirect (perte de productivité) élevés [1]. Selon la littérature, des cervicalgies sont associés à des facteurs de risques multiples, parmi lesquels les contraintes biomécaniques occupent une place centrale [1, 5, 44]. Notre actuel travail a objectivé par des observations directes et les études anthropométriques au non-respect des normes ergonomiques dans les postes de travail sur écran des 325 opérateurs exerçant dans les 25 institutions universitaires inclus dans l'étude. L’analyse semi-quantitative a permis d’objectiver l’association de ces manquement aux recommandations ergonomiques à multiples contraintes biomécaniques notamment à type de flexion franche du cou plus des deux tiers du temps global du travail et une flexion latérale plus de la moitié du temps de travail. Nos résultats impliquent le besoin d’une optimisation de l'organisation du travail et de conception ergonomique des postes de travail pour mieux prévenir les cervicalgies.

### Etat des connaissances actuelles sur le sujet

- Les cervicalgies sont un problème fréquent en milieu de travail avec un coût élevé pour l'individu et pour la société;
- Les contraintes biomécaniques associées au travail sur écran sont des forts prédicateurs du développement des cervicalgies.

### Contribution de notre étude à la connaissance

- Le non-respect des normes ergonomique est fort noté parmi les opérateurs sur écran dans les établissement universitaires tunisiens avec notamment des sièges et des plans de travail inadaptés, des écran bas situés par rapport au regard horizontal et dans un local insuffisamment éclairé;
- Multiples contraintes biomécaniques sont associées à la conception du poste de travail sur écran non conforme aux dimensions anthropométriques ergonomiquement recommandées notamment de type de flexion franche et une rotation latérale du cou lors de l’exercice de plusieurs tâches professionnelles.

## Conflits d’intérêts

Les auteurs ne déclarent aucun conflit d'intérêts.
